# Microstructure Evolution in Cu-Ni-Co-Si-Cr Alloy During Hot Compression by Ce Addition

**DOI:** 10.3390/ma13143186

**Published:** 2020-07-16

**Authors:** Yijie Ban, Yi Zhang, Baohong Tian, Yanlin Jia, Kexing Song, Xu Li, Meng Zhou, Yong Liu, Alex A. Volinsky

**Affiliations:** 1 School of Materials Science and Engineering, Henan University of Science and Technology, Luoyang 471023, China; byj18039620621@163.com (Y.B.); bhtian007@163.com (B.T.); zhoumeng0902@126.com (M.Z.); lyly2008@163.com (Y.L.); 2 Provincial and Ministerial Co-Construction of Collaborative Innovation Center for Non-Ferrous Metal New Materials and Advanced Processing Technology, Luoyang 471023, China; 3 Henan Province Key Laboratory of Nonferrous Materials Science and Processing Technology, Luoyang 471023, China; 4 College of Materials Science and Engineering, Beijing University of Technology, Beijing 100124, China; 5 Center for Advanced Measurement Science, National Institute of Metrology, Beijing 100029, China; li-xu@nim.ac.cn; 6 Department of Mechanical Engineering, University of South Florida, Tampa, FL 33620, USA; volinsky@usf.edu

**Keywords:** Cu-Ni-Co-Si-Cr-(Ce) alloys, hot deformation, microstructure evolution, electron backscattered diffraction (EBSD), processing map

## Abstract

Cu-Ni-Si alloys are widely used in lead frames and vacuum devices due to their high electrical conductivity and strength. In this paper, a Cu-Ni-Co-Si-Cr-(Ce) alloy was prepared by vacuum induction melting. Hot compression tests of the Cu-Ni-Co-Si-Cr and Cu-Ni-Co-Si-Cr-Ce alloys were carried out using a Gleeble-1500 simulator at 500–900 °C deformation temperatures and 0.001–10 s^−1^ strain rates. The texture change was analyzed by electron backscatter diffraction. The <110> fiber component dominated the texture after compression, and the texture intensity was reduced during recrystallization. Moreover, the average misorientation angle φ for Cu-Ni-Co-Si-Cr-Ce (11°) was lower than that of Cu-Ni-Co-Si-Cr (16°) under the same conditions. Processing maps were developed to determine the optimal processing window. The microstructure and precipitates of the Cu-Ni-Co-Si-Cr and Cu-Ni-Co-Si-Cr-Ce alloys were also analyzed. The average grain size of the Cu-Ni-Co-Si-Cr-Ce alloy (48 μm) was finer than that of the Cu-Ni-Co-Si-Cr alloy (80 μm). The average size of precipitates in the Cu-Ni-Co-Si-Cr alloy was 73 nm, while that of the Cu-Ni-Co-Si-Cr-Ce alloy was 27 nm. The addition of Ce delayed the occurrence of dynamic recrystallization.

## 1. Introduction

High electrical conductivity functional materials have been widely used in lead frames, in the electronics industry, and in electrical vacuum devices. Copper alloys are widely used because of their high conductivity and strength. Vacuum devices, including ultrahigh frequency emission tubes, waveguide tubes, and magnetron tubes have high performance requirements. Thus, high-purity, oxygen-free copper and dispersion strengthened oxygen-free copper are good choices for these applications. In the past few years, Cu-Ni-Si alloy has become one of the most rapidly developing copper alloys [1,2,3,4,5,6]. Trace elements, such as Co [7], P [8], Cr [9,10], Ag [11], Ti [12], and so on, have been added to further improve its properties. Krishna et al. [13] found that Ni_2_Si precipitation can improve the strength of Cu-Ni-Si alloys. Co can also effectively improve performance. It is worth noting that Co and Ni have similar atomic radii, and a small amount of Co can replace Ni to form (Ni, Co)_2_ Si precipitates, as confirmed by Zhao et al. [14]. Cu-Ni-Si alloys have been researched continuously for many years. Wang et al. [15] prepared a Cu-Ni-Si-Cr alloy by hot pressing and sintering, studying prior particle boundary defects and eliminating residual porosity in the ingot.

The addition of rare-earth elements significantly improves the alloy properties. Rare-earth elements are widely used in electronics, petrochemicals, and so on, because of their excellent physical, chemical, magnetic, and electrical properties. The addition of appropriate amounts of rare-earth elements or compounds can improve plasticity, toughness, heat, and corrosion resistance. Rare-earth metals can be made into permanent magnetic materials, such as SmCo_5_, Sm_2_Co_17_, Sm_2_Fe_17_N_x_, and others. Adding some rare-earth metals to ceramic capacitor materials can improve their stability and service life. Stanford et al. [16] concluded that the addition of Ce could reduce the yield point and improve the ductility of the alloy. Zhang et al. [17,18] investigated the effects of the addition of Ce and Y on the evolution of the microstructure and precipitates in a Cu-Mg alloy during hot deformation, and found that it could significantly delay dynamic recrystallization and increase the flow stress.

Hot deformation of materials is the basis of thermal processing and is widely applied in manufacturing to improve alloy properties. Based on the above research, this experiment mainly explored the effects of Ce on the microstructure and recrystallization behavior of a Cu-Ni-Co-Si-Cr alloy during hot deformation. Hot compression tests were carried out by using a Gleeble-1500 simulator at 0.001–10 s^−1^ strain rates and 500–900 °C deformation temperatures. The effects of the addition of Ce on the evolution of the microstructure were studied by comparing transmission electron microscopy (TEM) images. Also, the effect of the addition of Ce on the dislocation density and texture changes were investigated by electron backscatter diffraction (EBSD).

## 2. Experimental 

The Cu-Ni-Co-Si-Cr and Cu-Ni-Co-Si-Cr-Ce alloys used in this experiment were vacuum melted with 99.5 wt.% copper, pure Ni, Co, Si, and Cr, and Cu-19% Ce master alloys were prepared by vacuum induction melting. Table 1 shows the nominal and actual compositions of the two alloys. It was found that there were also trace amounts of C, N, O, and H in the alloy in addition to the main elements. These elements originated from the smelting process. Although vacuum treatment was carried out during the smelting process, a small number of hydrocarbons, O_2_, and CO_2_ remained in the furnace. Additionally, moisture in the atmosphere remains in the furnace, so a small amount of H was absorbed during the smelting process. These gases were unavoidable during the casting process. To minimize the quantity of impurity elements in the alloy, 0.05 MPa Ar was injected as a protective gas. A copper block was cleaned and dried before melting. The obtained ingot was cooled at room temperature and the oxidized part was peeled. The ingots were annealed at 1000 °C for 1 h, and then extruded into a tubing of 35 mm diameter using an XJ-500 metal profile extrusion machine (Wuxi Yuanchang Machinery Co., Ltd., Jiangsu, Wuxi, China). The obtained samples were solution treated in a KSS-1200 tubular resistance furnace (Hengsu technology, Zhengzhou, China) at 950 °C for 2 hours, and then water-cooled. Finally, the samples were cut into 8 mm × 12 mm cylinders for hot compression testing.

Hot compression experiments were carried out on a Gleeble-1500D thermo-mechanical simulator (Dynamic Systems Inc., Rensselaer County, NY, USA). The cylindrical specimens were isothermally compressed at 500 °C to 900 °C with 100 °C intervals at strain rates of 0.001 s^−1^, 0.01 s^−1^, 0.1 s^−1^, 1 s^−1^, and 10 s^−1^. The compression of the samples was 55%. The samples were heated to a given temperature with a heating rate of 10 °C/s and then kept at this temperature for three minutes. After hot compression, the samples were quenched in water immediately to maintain the deformation structure.

The microstructure was observed using an OLYMPUS PMG3 optical microscope (Olympus Corporation, Tokyo, Japan), a JMS-7800F field emission scanning electron microscope (Hitachi, Tokyo, Japan, SEM), and a JEM-2100 transmission electron microscope (Jeol, Tokyo, Japan). The samples for EBSD analysis were acquired from the central part of the samples along the compression direction, and EBSD maps (241 × 166 μm^2^) were measured using a 1-μm step size. The indexing rate was 95%. The software Transmission Channel 5 was used to analyze the EBSD data. A minimum misorientation angle of 1° was used to detect individual crystallites. The nonrecrystallized grains were distinguished from the recrystallized ones by the Grain orientation spread (GOS). For the TEM samples preparation, the specimen was processed into a 50-μm thick wafer with a 3 mm diameter, and subsequently ion thinned using a Gatan 691 (Gatan, Pleasanton, CA, USA) ion beam thinner. 

## 3. Results

### 3.1. Microstructure Evolution

**Figure 1:**
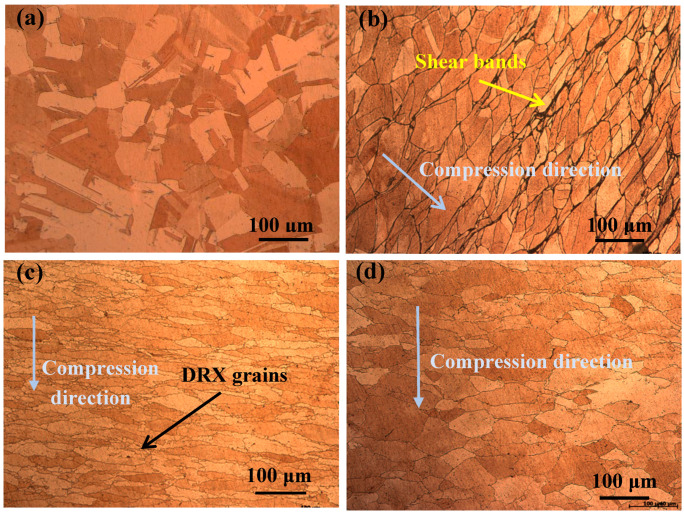
Optical images of the Cu-Ni-Co-Si-Cr alloy microstructure deformed under different conditions: (**a**) solid-solution; (**b**) 500 °C and 0.01 s^−1^; (**c**) 700 °C and 0.01 s^−1^; and (**d**) 900 °C and 0.01 s^−1^.

**Figure 2:**
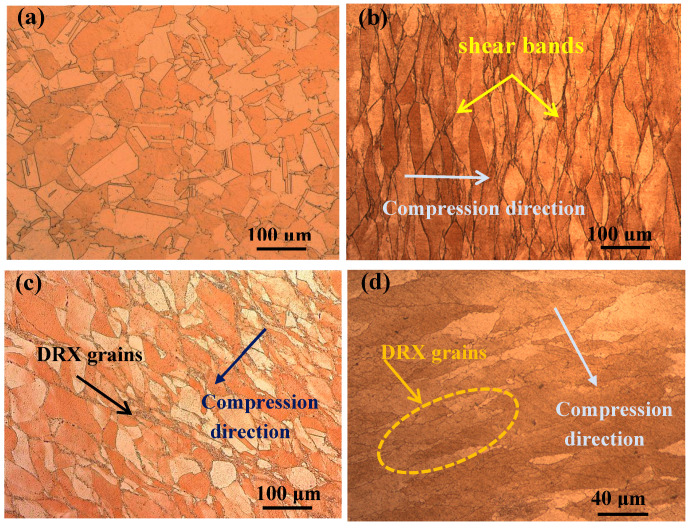
Optical images of the Cu-Ni-Co-Si-Cr-Ce alloy microstructure deformed under different conditions: (**a**) solid-solution; (**b**) 500 °C and 0.01 s^−1^; (**c**) 700 °C and 0.01 s^−1^; and (**d**) 900 °C and 0.01 s^−1^.

Figure 1 and Figure 2 show the microstructure of the Cu-Ni-Co-Si-Cr and Cu-Ni-Co-Si-Cr-Ce alloys. The microstructure of the solid solution is shown in Figure 1a and Figure 2a. The average grain size of the Cu-Ni-Co-Si-Cr-Ce alloy was much smaller than that of the Cu-Ni-Co-Si-Cr alloy, i.e., 48 μm and 80 μm, respectively. This indicates that the addition of Ce can refine grains, and when the grain size is refined, the strength and toughness of the alloy are improved. Figure 1b shows the microstructure of the Cu-Ni-Co-Si-Cr alloy deformed at 500 °C and 0.01 s^−1^. Due to the low deformation temperature, the original grains were elongated and shear bands appeared, primarily at grain boundaries. This means that the stress was concentrated there, leading to work hardening [19,20,21]. With the increase in temperature, dynamic recrystallization occurred, as shown in Figure 1c. It is worth noting that the recrystallized grains mainly formed at the grain boundary. Because of the high distortion energy at the grain boundaries, the recrystallized grains gradually replaced the original grains [22]. Compared with the Cu-Ni-Co-Si-Cr-Ce alloy deformed under the same conditions (Figure 2c), the average size of the recrystallized grains in the Cu-Ni-Co-Si-Cr alloy was 9 μm, while that of the Cu-Ni-Co-Si-Cr-Ce alloy was much smaller.

Figure 1d shows the microstructure of the Cu-Ni-Co-Si-Cr alloy deformed at 900 °C and 0.01 s^−1^. With the increased degree of recrystallization, the recrystallized grains coarsened and finally replaced the original grain structure. However, fine dynamic recrystallization grains still existed in the Cu-Ni-Co-Si-Cr-Ce alloy, as seen in Figure 2d. This proves that the addition of Ce delays the dynamic recrystallization process.

### 3.2. Precipitates

**Figure 3:**
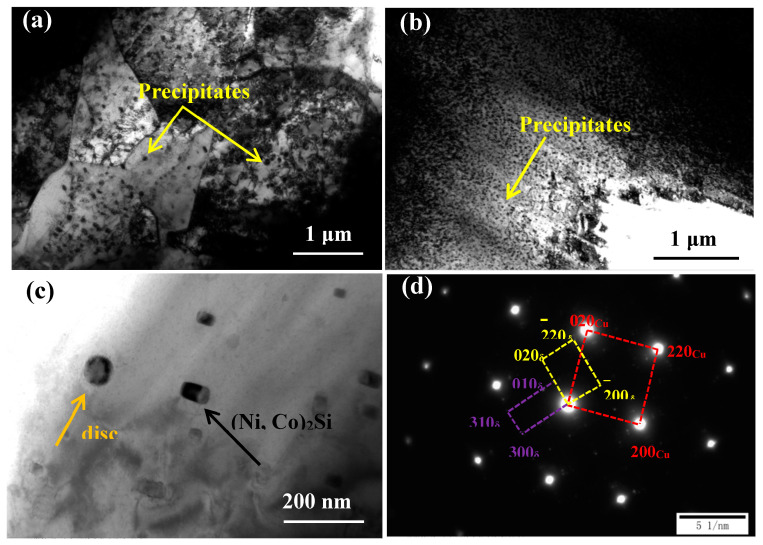
TEM micrographs of samples deformed at 700 °C and 0.01 s^−1^: (**a**) Cu-Ni-Co-Si-Cr; (**b**,**c**) Cu-Ni-Co-Si-Cr-Ce; (**d**) SADP of corresponding area in (**c**).

Figure 3a,b shows TEM micrographs of the Cu-Ni-Co-Si-Cr and Cu-Ni-Co-Si-Cr-Ce alloys deformed at 700 °C and 0.01 s^−1^. The number of precipitates increased significantly after the addition of Ce. Also, the average size of the precipitates in the Cu-Ni-Co-Si-Cr alloy was 73 nm, while that of the Cu-Ni-Co-Si-Cr-Ce alloy was 27 nm. According to the above analysis, the strength of the alloy improved after the addition of Ce. Figure 3c shows the shape of precipitates in the alloy. They can be divided into disc- and rod-shaped forms, as expected for (Ni, Co)_2_Si; the existence of (Ni,Co)_2_Si has been confirmed by many scholars [23,24,25]. Co and Ni have similar atomic radii. The lattice parameters of Ni_2_Si are a = 0.708 nm, b = 0.490 nm, and c = 0.373 nm, with Pbnm space group 62 [26]. The lattice parameters of Co_2_Si are a = 0.710 nm, b = 0.491 nm, and c = 0.377 nm [27]. Therefore, some of the Ni atoms in the Ni_2_Si phase will be replaced by Co atoms, forming (Ni,Co)_2_Si. At the same time, the corresponding selected area diffraction patterns (SADP) in Figure 3d show that the spots are derived from the electron diffraction of (Ni,Co)_2_Si.

### 3.3. EBSD

**Figure 4:**
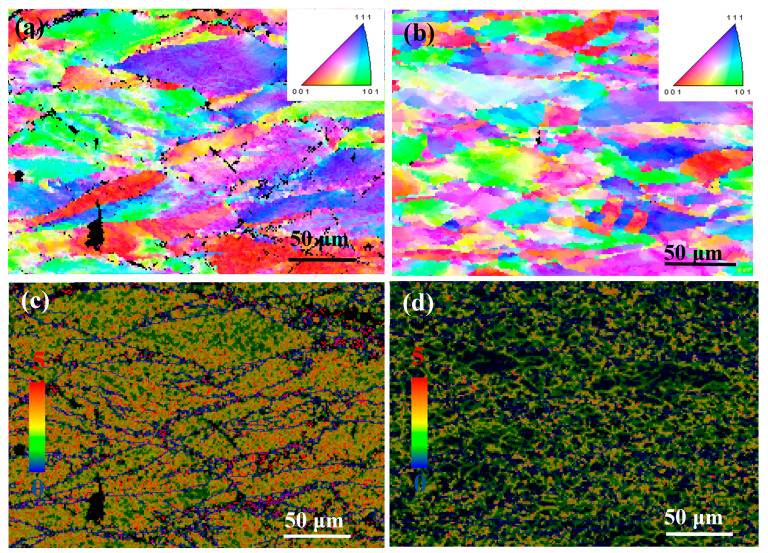
EBSD micrographs and Kernel Average Misorientation of the Cu-Ni-Co-Si-Cr alloy deformed at: (**a**,**c**) 600 °C and 0.001 s^−1^, (**b**,**d**) 900 °C and 0.001 s^−1^.

**Figure 5:**
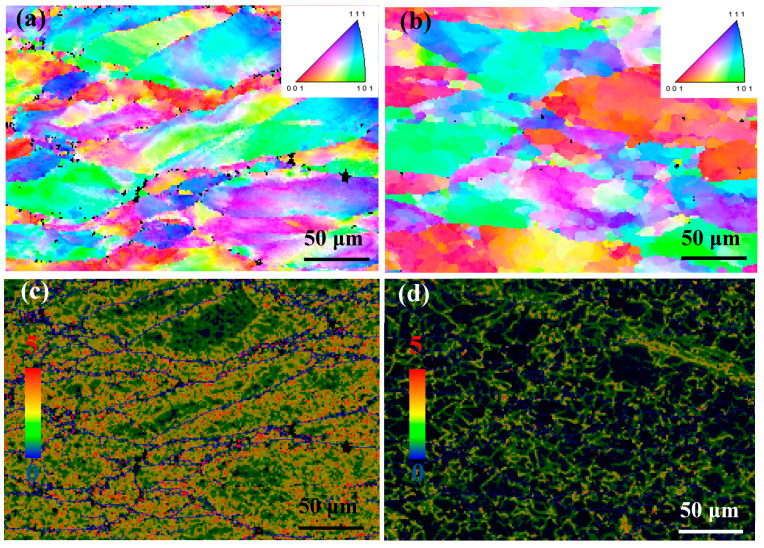
EBSD micrographs and Kernel Average Misorientation of the Cu-Ni-Co-Si-Cr-Ce alloy deformed at: (**a**,**c**) 600 °C and 0.001 s−1, (**b**,**d**) 900 °C and 0.001 s−1.

Figure 4 and Figure 5 show the EBSD micrographs of the Cu-Ni-Co-Si-Cr and Cu-Ni-Co-Si-Cr-Ce alloys, respectively. At a low deformation temperature (Figure 4a), the hot compression made the grains elongated, and the recrystallized grains existed at the grain boundary, but the degree of recrystallization was very low (the number fraction is 21%). With the increase of temperature (Figure 4b), the original grains were gradually replaced by the recrystallized grains (the number fraction was 58%). Figure 4c,d show the dislocation density of the Cu-Ni-Co-Si-Cr alloy at 600 °C and 900 °C, respectively. The red region represents high dislocation density, while the blue one represents low dislocation density. The migration of grain boundaries plays an important role in hot deformation, and the migration of grain boundaries is related to dislocations near the grain boundaries. 

From the kernel average misorientation (KAM) maps, it can be seen that the average KAM value at 600 °C of 2.03 was higher than 1.467 at 900 °C, indicating the occurrence and coarsening of dynamic recrystallization [28,29]. Similarly, this phenomenon was also found in the Cu-Ni-Co-Si-Cr-Ce alloy, as shown in Figure 5c,d respectively.

**Figure 6:**
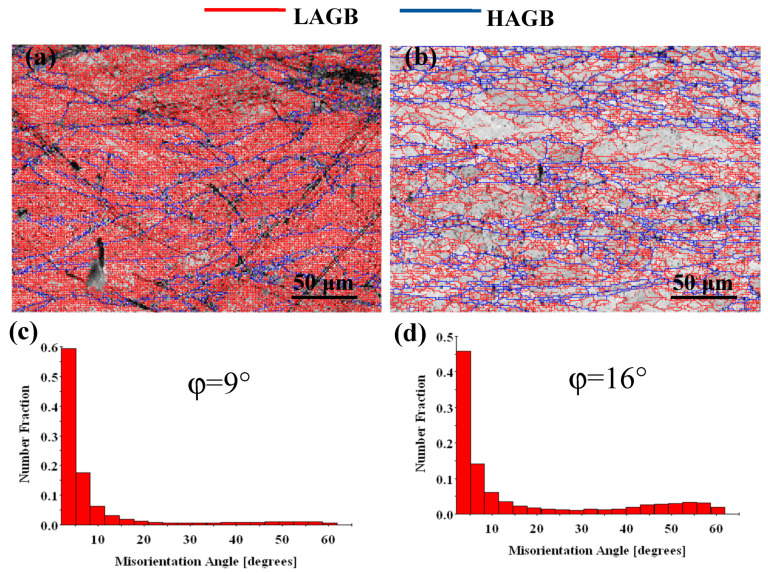
Orientation maps and misorientation angle distributions of the Cu-Ni-Co-Si-Cr alloy deformed at: (**a**,**c**) 600 °C, 0.001 s^−1^; (**b**,**d**) 900 °C, 0.001 s^−1^. HAGBs are shown as blue lines, and LAGBs are shown as red lines. φ: The average misorientation angle.

**Figure 7:**
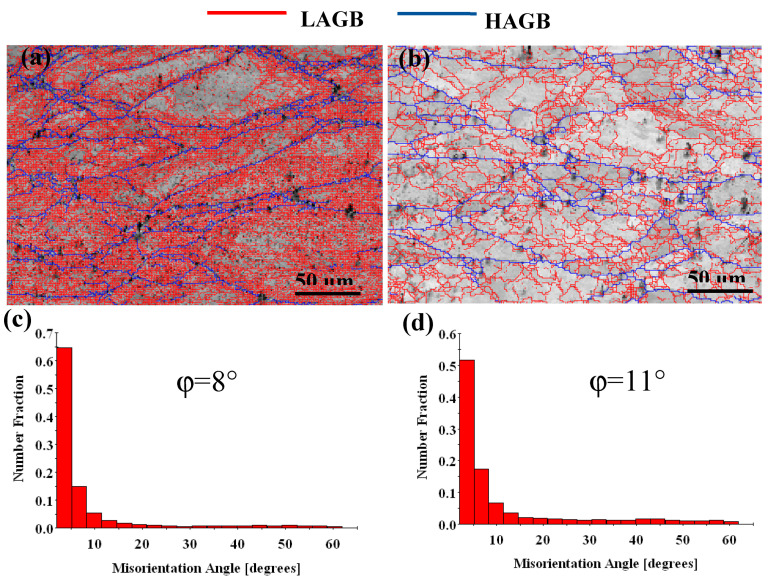
Orientation maps and misorientation angle distributions of the Cu-Ni-Co-Si-Cr-Ce alloy deformed at: (**a**,**c**) 600 °C, 0.001 s^−1^; (**b**,**d**) 900 °C, 0.001 s^−1^. HAGBs are shown as blue lines, and LAGBs are shown as red lines. φ: The average misorientation angle.

Figure 6 and Figure 7 show the orientation maps and misorientation angle of the Cu-Ni-Co-Si-Cr and Cu-Ni-Co-Si-Cr-Ce alloys. In the orientation maps, the grain boundaries with a misorientation angle higher than 15° may be defined as high angle grain boundaries (HAGBs), while those with a misorientation angle lower than 15° are called low angle grain boundaries (LAGBs) [30,31]. In the process of hot deformation, many low angle grain boundaries occur at low temperature (Figure 6a), which results in the aggregation of dislocations in the deformed grain boundaries and work hardening regions. With the increase in temperature, recrystallization occupies the dominant position. The recrystallized grains grow and coarsen, transforming the low angle grain boundaries into high angle grain boundaries. The average misorientation angle φ for the Cu-Ni-Co-Si-Cr alloy increased from 9° to 16°, as shown in Figure 6c,d. The percentage of high angle grain boundaries increased from 12% to 29% with temperature.

Compared with the Cu-Ni-Co-Si-Cr-Ce alloy produced using the same method, the average misorientation angle φ increased from 8° to 11°, as shown in Figure 7c,d, and the percentage of high angle grain boundaries increased from 11% to 19% with temperature. The average misorientation angle and the high angle grain boundaries of the Cu-Ni-Co-Si-Cr-Ce alloy were smaller than those of the Cu-Ni-Co-Si-Cr alloy. Therefore, it can be concluded that the addition of Ce delayed the dynamic recrystallization of the Cu-Ni-Co-Si-Cr alloy.

**Figure 8:**
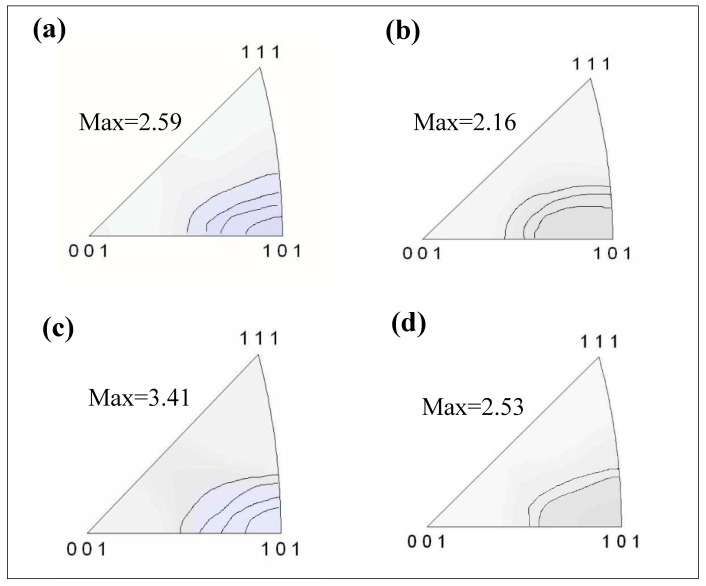
Textures in the Cu-Ni-Co-Si-Cr alloy and Cu-Ni-Co-Si-Cr-Ce alloy represented by inverse pole figures for the compression direction. (**a**,**b**) Cu-Ni-Co-Si-Cr alloy deformed at 600 °C, 0.001 s^−1^ and 900 °C, 0.001 s^−1^, respectively; (**c**,**d**) Cu-Ni-Co-Si-Cr-Ce alloy deformed at 600 °C, 0.001 s^−1^ and 900 °C, 0.001 s^−1^, respectively.

The textures of the Cu-Ni-Co-Si-Cr and Cu-Ni-Co-Si-Cr-Ce alloys are demonstrated in Figure 8. It is well known that the texture is typically <110> fiber for face centered cubic (fcc) metals after compression [32]. The dominant texture obtained in this work was also <110> fiber, which was consistent with the research results of Chen. et al. [33]. However, it should be noted that the texture intensity reduced during recrystallization.

### 3.4. Peak Stress

**Figure 9:**
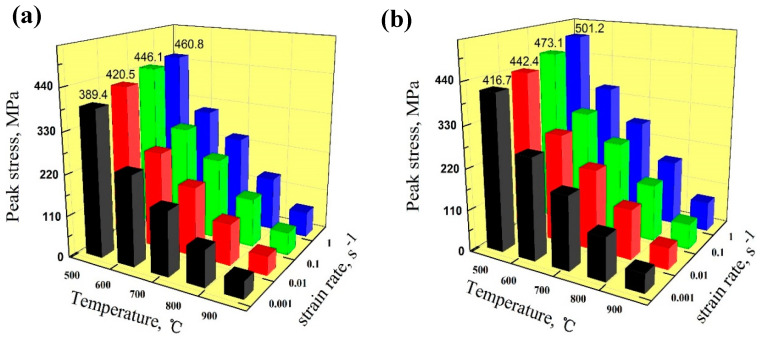
The peak stress at different temperatures and strain rates: (**a**) Cu-Ni-Co-Si-Cr alloy; (**b**) Cu-Ni-Co-Si-Cr-Ce alloy.

Figure 9 shows the peak stress of the Cu-Ni-Co-Si-Cr and Cu-Ni-Co-Si-Cr-Ce alloys deformed at different strain rates and temperatures. During the hot deformation process, the alloy reached peak stress due to work hardening; there were a lot of dislocation entanglements at this stage. The peak stress increased with decreased temperature or increased strain rate. This was because the kinetic energy of atoms increases with temperature, which makes dislocation movement and thermal diffusion more active. Moreover, at the low strain rate, the increase of dislocation density was relatively gentle, which reduced the alloy’s peak stress [34,35,36,37,38].

The peak stress increased after the addition of Ce; the maximum peak stress was 501 MPa. According to the above analysis, the peak stress increase was due to the refining effect of the addition of Ce on the grains. Furthermore, the addition of Ce promoted precipitation and reduced the precipitate size. Eventually, the peak stress of the Cu-Ni-Co-Si-Cr-Ce alloy increased.

### 3.5. Processing Map

To further study the effects of Ce on the hot deformation of the alloy, hot processing maps were established based on the dynamic materials model (DMM). In this model, the workpiece is regarded as a power dissipater. The total power *P* in the process of system deformation can be considered as two harmonizing parts [39,40]:

(1) $$P=\sigma \dot{\varepsilon }=G+J=\int_{0}^{\dot{\varepsilon }}\;\sigma d\dot{\varepsilon }+\int_{0}^{\sigma }\dot{\varepsilon }d\sigma$$

where *G* represents the power dissipation due to plastic deformation, and *J* the power dissipation associated with the change of microstructure. The fraction of power which is absorbed by deforming the material during hot processing is called the efficiency of power dissipation, *η*. *η* is related to *J* as:

(2) $$\eta =\frac{J}{J_{\max}}=\frac{2m}{m+1}$$

where m is the strain rate sensitivity parameter:

(3) $$m=\frac{\partial \;J}{\partial G}=\frac{\partial \ln\sigma }{\partial \ln\dot{\varepsilon }}$$

The values of *η*, *T*, and *έ* constitute the power dissipation map and reflect changes in the microstructure. The power dissipation map shows the region with the highest power efficiency. The higher the value of *η*, the better the workability that can be obtained. Meanwhile, an instability map of the alloy can be obtained according to the Prasad flow instability criterion [41]:

(4) $$\xi \left(\dot{\varepsilon }\right)=\frac{\partial \ln\left(\frac{m}{m+1}\right)}{\partial \ln\dot{\varepsilon }}+m<0$$

The instability map was constituted of variations of the instability parameter, temperature, and strain rate. The processing map was constructed by superimposing the instability map over the power dissipation map.

**Figure 10:**
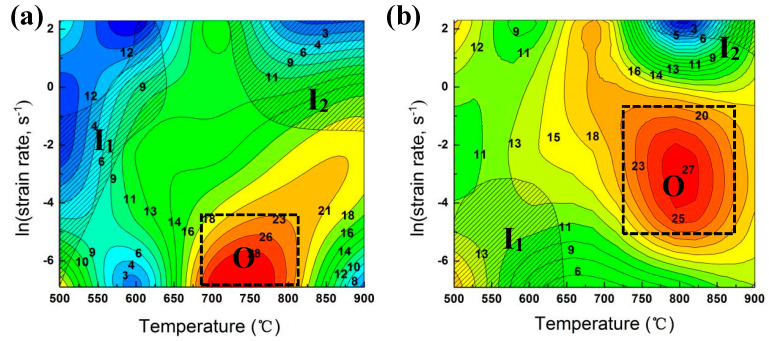
Hot processing maps of: (**a**) Cu-Ni-Co-Si-Cr and (**b**) Cu-Ni-Co-Si-Cr-Ce alloy. The shaded areas represent instability domain. The boxes represent the optimal processing domain. **O**: Optimal Processing Domain. **I**: Instability Domain.

Figure 10 shows the processing map of the Cu-Ni-Co-Si-Cr and Cu-Ni-Co-Si-Cr-Ce alloys at a true strain of 0.35. The values in the figure represent the efficiency of power dissipation. The colored and shaded areas correspond to secure and instability domains, respectively.

Comparing the domain O (optimal processing domain) of the two alloys, it can be seen that the higher power dissipation value region of the Cu-Ni-Co-Si-Cr alloy was located at 650–800 °C with strain rates of 0.001–0.02 s^−1^. The domain O of the Cu-Ni-Co-Si-Cr-Ce alloy was mainly located at 700–900 °C with strain rates of 0.006–0.4 s^−1^. Dynamic recrystallization was the main process of the alloy at this time, and the recrystallization grain size was relatively uniform.

At a lower deformation temperature, the work hardening causes dislocation entanglement, resulting in the uneven distribution of stress in the structure. At this stage, it has the characteristics of elongated grains and shear bands, which are prone to cracking. Meanwhile, there was not enough time for the heat generated by deformation to conduct to the colder part under a high strain rate, which led to an uneven temperature distribution in the deformed specimen. Therefore, a high strain rate or low temperature constitute the instability domain of a hot working process and should be avoided.

It is worth noting that the unstable region of the alloy decreases after adding Ce. The processing map created using the dynamic material model provides theoretical data for the actual processing conditions, reducing waste and pollution.

## 4. Conclusions

Hot compression tests of Cu-Ni-Co-Si-Cr and Cu-Ni-Co-Si-Cr-Ce alloys were conducted at 0.001–10 s^−1^ strain rates and 500–900 °C deformation temperatures. The effects of the addition of Ce on the evolution of the microstructure and precipitates were discussed, and the following conclusions were drawn:

1. The <110> fiber component dominated the texture of the Cu-Ni-Co-Si-Cr and Cu-Ni-Co-Si-Cr-Ce alloys after compression; it should be noted that the texture intensity reduced during recrystallization.
2. The (Ni, Co)_2_Si precipitate was found during the hot compression process, while precipitates in the Cu-Ni-Co-Si-Cr-Ce alloy (27 nm) were finer than in the Cu-Ni-Co-Si-Cr alloy (73 nm).
3. The addition of Ce refined the grain and delayed dynamic recrystallization.
4. The peak stress increased with decreased temperature or increased strain rate. The addition of Ce increased the peak stress of the Cu-Ni-Co-Si-Cr alloy; the maximum peak stress was 501 MPa.
5. Based on the dynamic material model, processing maps were established for Cu-Ni-Co-Si-Cr and Cu-Ni-Co-Si-Cr-Ce alloys. The optimal processing parameters of the former were 650–800 °C with strain rates of 0.001–0.02 s^−1^, while for the latter, they were 700–900 °C with strain rates of 0.006–0.4 s^−1^. It is worth noting that the unstable region of the alloy decreased after adding Ce, which indicates that the addition of Ce improves the hot processing properties of the alloy.

## Figures and Tables

**Table 1:** The nominal and analyzed compositions of the alloys.

Alloy	Analyzed Composition(wt.%)				
	Ni	Co	Si	Cr	Cu
Cu-1.5Ni-1.1Co-0.6Si-0.1Cr	1.43	1.02	0.54	0.93	Bal.
Cu-1.5Ni-1.1Co-0.6Si-0.1Cr-0.15Ce	1.39	1.04	0.52	0.89	Bal.
	Ce	N	C	O	H
	0.14	0.13	0.06	0.09	0.04
